# Energetics and reactivity of small beryllium deuterides

**DOI:** 10.1007/s00894-017-3362-4

**Published:** 2017-06-16

**Authors:** Ivan Sukuba, Alexander Kaiser, Stefan E. Huber, Jan Urban, Michael Probst

**Affiliations:** 1 Institute of Ion Physics and Applied Physics, University of Innsbruck, Technikerstraße 25, A-6020 Innsbruck, Austria; 2 Department of Nuclear Physics and Biophysics, Faculty of Mathematics, Physics and Informatics, Comenius University, SK-84248 Bratislava, Slovakia

**Keywords:** Beryllium deuterides, Dissociation, ITER, Reactivity, Quantum-chemical calculations, Molecular dynamics

## Abstract

Enthalpies and free energies of reaction for small neutral and charged beryllium deuterides BeD, BeD_2_, and BeD_3_ that have been calculated are reported for a temperature range of 0 K to 1000 K. We discuss probable dissociation channels and possible ways of producing BeD by localizing the relevant transition states and by calculating corresponding rate constants. BeD and BeD^+^ are found to be the most stable ones among the considered compounds. BeD_2_ and ${\mathrm{BeD}}_2^{+}$ are more likely to decompose into Be^0,+^ + D_2_ than into BeD^0,+^ + D. The metastable BeD_3_ and ${\mathrm{BeD}}_3^{+}$ predominantly decompose into BeD^0,+^ + D_2_. In light of our results on the reaction energetics, we can interpret the pathways for production of BeD via BeD_2_ and BeD_3_ intermediates observed in molecular dynamics simulations.

## Introduction

The development of new technologies for controlled fusion caused beryllium compounds, especially hydrides that can be created by D/T bombardment from plasma, to become one focus of materials research. In the ITER reactor, beryllium is planned to be the first-wall material, and hence it will be directly exposed to particles, predominantly deuterons, that escape the confinement as is already observed in the JET tokamak with ITER-like walls [1, 2]. For many years, plasma-wall interactions (PWI) have been extensively studied experimentally as well as theoretically. The main source of experimental data concerning beryllium-deuterium interactions are linear devices like PISCES-B [3, 4] or tokamaks [5–7]. However, the underlying processes like sputtering, transport and deposition are hard to reproduce and quantify experimentally. Modeling and theoretical approaches to obtain data for codes like Wall-DYN [8], ERO [9] or SDTrimSP [10, 11] are therefore developed to get insight into such processes. Theoretical studies of plasma wall interactions involve the description of the interaction of surfaces with the fusion plasma [12], the characterization of elementary processes [13] as well as the validation of experimental results [14, 15]. Experimental results of plasma-surface interactions confirm the complexity of the whole process. Concerning beryllium experiments, there is evidence of the formation of BeD molecules and of a linear drop of the BeD:Be ratio with increasing temperature in the temperature range of 500–700 K while no larger molecules like BeD_2_ or BeD_3_ were observed [7]. In contrast, molecular dynamics (MD) simulations employing analytical bond-order potentials (ABOP) [16, 17], as well as multiscale modeling extrapolated from them [18] predict BeD_2_ and BeD_3_ as the main eroded species for the same temperature range [19]. Since at lower temperatures (<500 K) MD simulations were in agreement with experiment, a more complete description of the possible fragmentation processes is needed from both the energetic and kinetic points of view. Dissociation and reactivity of beryllium hydrides and their isotopes were briefly discussed by Safi et al. [19] and Virot et al. [20] on the basis of standard thermodynamic data and possible reaction channels were given for the dissociation of BeD_2_ and BeD_3_ at various temperatures. The dissociation and ionization rates for primary reactions of BeD due to electron collisions were reported by Björkas et al. [18]. In the present work, we employ quantum chemical methods including comparisons between various levels of theory and we study the reaction kinetics to determine the reactivity of different channels. Highly accurate data have been published before on the beryllium hydrogen systems: BeH as well as BeH_2_ have received a significant amount of interest as a test system for quantum chemical methods including non-standard ones [21–29]. The multi-reference averaged coupled-pair functional method (MR ACPF) [21] was used to calculate the accurate ground state potential energy functions, vibration-rotation energy levels for BeH, BeD, and BeT and their ions, which agree excellently with spectroscopic experimental data, i.e., the equilibrium bond length R_e_ = 1.341 Å [22, 23]. Non-Born-Oppenheimer variational calculations employing explicitly correlated Gaussian basis functions were performed in order to determine the ionization energy of BeH and the dissociation energies of BeH and BeH^+^ [24]. Penotti’s [26] non-orthogonal single and multi-configurational calculations with a highly optimized even-tempered STO basis set yielded a value of R_e_ = 1.329 Å for the D_∞h_ geometry of the BeH_2_ molecule. A value of 2053.0 cm^−1^ was obtained for the harmonic symmetric-stretch frequency. Very precise results for the BeH system also included non-adiabatic effects and extrapolation of the basis set up to the *spdfgh* level as well as extrapolation of correlation effects to the full configuration interaction (FCI) limit [28]. An equilibrium distance of R_e_ = 1.341 Å and a ground state frequency of ω_e_ = 2062.1 cm^−1^ were reported. Hinze et al. [29] published potential energy surfaces (PESs) for BeH_2_ and ${\mathrm{BeH}}_{\mathbf{2}}^{+}$ obtained with the multi-reference configuration interaction method (MRCI) and documented the insertion reaction of Be into H_2_. Koput and Peterson [30] obtained vibrational and rotational energy levels of beryllium dihydride and of its isotopes from an accurate potential energy surface using CCSD (T) and extrapolation to the full basis set limit. The IR emission spectra for BeH and BeH_2_ were measured by Bernath and coworkers [31–34]. They obtained R_e_ = 1.342 Å, ω_e_ = 2061.4 cm^−1^ for BeH, and R_e_ = 1.326 Å, ω_e_ = 2255.2 cm^−1^ for the asymmetric stretch for BeH_2_. Reaction enthalpies for the dissociation channels of the BeD_3_ molecule were calculated in ref. [19] and the thermodynamic stability of neutral and anionic BeH_3_ was analyzed in ref. [35]. The knowledge of the whole reaction network is necessary for understanding the chemical behavior of the Be/H system. This work aims to describe the fragmentation and reactivity of small beryllium deuterides in the temperature range 0–1000 K and is based on quantum-chemistry calculations and transition state theory. We report a stability analysis, thermodynamic data of neutral and charged BeD_1–3_ molecules, the standard enthalpies and free energies of reaction for their possible dissociation channels, and their corresponding dissociation energies. Calculated transition states and activation energies can be used to estimate reaction rate constants. Furthermore, reaction schemes for the production of BeD from beryllium surfaces exposed to D irradiation as extracted from MD simulations are discussed.

## Computational methods

### Quantum-chemical calculations

The optimized structures and vibrational frequencies of the beryllium hydrides were obtained by the Gaussian-4 (G4) [36] method and by density functional theory (DFT). We compared different DFT functionals: the often used hybrid functional B3LYP-D [37, 38], the B97D functional and the meta-GGA M06 functional [39]. The first two functionals contain Grimme’s GD3 empirical dispersion parameters [40]. We also employed the double hybrid B2PLYPD [41, 42] functional which includes dispersion and density corrections by second-order Møller–Plesset perturbation theory (MP2) [43]. Furthermore, we optimized the predicted structures by coupled cluster calculations with single and double substitutions and non-iteratively included triple excitations (CCSD(T)) [44] with all electrons correlated. All calculations were performed with Dunning’s correlation consistent core-valence quadruple zeta (aug-cc-pCVQZ) basis set [45], the sole exception being the pre-defined G4 method, and employed the GAUSSIAN 09 software package [46]. Different scaling factors are recommended for some of the methods used throughout this study [47]. However, we report unscaled frequencies here with the intention to introduce as little empiricism as possible.

### Thermodynamics

In order to qualitatively examine the reactivity of small beryllium deuterides (hydrides), we first calculated the standard enthalpies and the standard free energies of the reactants and products of the dissociation channels (Eq. 1). The standard enthalpies (the free energies) of reaction were calculated as a difference of the sum of electronic energy ε_0_, zero-point energy ϵ_ZPE_ and thermally corrected enthalpies (free energies) of products and reactants [48]:

1 $$ {\varDelta}_r{H}^{{}^{\circ}}\left(\mathrm{T}\right)=\sum_{products}\left(\ {\upvarepsilon}_0+{\epsilon}_{ZPE}+{H}_{corr}\left(\mathrm{T}\right)\right)-\sum_{r eactants}\left(\ {\upvarepsilon}_0+{\epsilon}_{ZPE}+{H}_{corr}\left(\mathrm{T}\right)\right) $$

and similarly for ∆_r_G^°^ (T). Only calculated values were used to obtain ∆_r_H^°^ and ∆_r_G^°^. Subsequently, equilibrium constants, K_EQ_, were obtained from the standard free energies of reactions:

2 $$ {\mathrm{K}}_{\mathrm{EQ}\ }(T)=\kern0.5em {e}^{\frac{-{\varDelta}_r{G}^{{}^{\circ}}(T)}{RT}} $$

The standard enthalpies of formation of molecules at 298.15 K, ∆_*f*_H^°^(M), were calculated using experimental enthalpies of formation for elements, ${\Delta}_{\mathrm{f}}{\mathrm{H}}_{0 K}^{{}^{\circ}}$ (X) and their corresponding thermal corrections, ${\mathrm{H}}_{corr}^{0\to 298.15},$ (see Table 1) and the procedure suggested by McQuarrie [48]:

3.a $$ {\varDelta}_f{\mathrm{H}}^{{}^{\circ }}\left(\mathrm{M}\right)={\varDelta}_f{{\mathrm{H}}^{{}^{\circ}}}_{0 K}\kern0.20em \left(\mathrm{M}\right)+{H}_{corr}^{0\to 298.15}\kern0.28em \left(\mathrm{M}\right)-\sum_{atoms} x{H}_{corr}^{0\to 298.15}\kern0.28em \left(\mathrm{X}\right) $$

3.b $$ {\varDelta}_f{{\mathrm{H}}^{{}^{\circ}}}_{0 K}\left(\mathrm{M}\right)=\sum_{atoms} x{\varDelta}_f{\mathrm{H}}_{0 K \mathit{^{\circ}}}^{{}^{\circ}}\left(\mathrm{X}\right)-{D}_0(M) $$

3.c $$ {D}_0(M)=\left(\sum_{atoms} x{\epsilon}_0(X)-{\epsilon}_0(M)-{\epsilon}_{ZPE}(M)\kern0.5em \right), $$

**Table 1:** Enthalpies of formation of elements in the gas state and thermal corrections to these enthalpies. Values are in kJ mol^-1^ rounded to two decimal points. Experimental and calculated ${\mathrm{H}}_{\mathrm{corr}}^{0\to 298.15}$ for hydrogen atoms were obtained from H_2_ (D_2_) values with the NIST-JANAF database [49]. The reference state for Be is a hcp crystal structure up to 1560 K, the calculated value refers to the gas state

	**Be**	**Be** ^**+**^	**H**	**H** ^**+**^	**D**	**D** ^**+**^
${\Delta}_{\boldsymbol{f}}{\boldsymbol{H}}_{0\boldsymbol{K}{}^{\circ}}^{{}^{\circ}}$	319.74	1219.23	216.04	1488.36	219.77	1492.29
${\boldsymbol{H}}_{\boldsymbol{corr}}^{0\to 298.15}$ **exp.**	1.93	6.20	4.23	4.29	4.29	4.33
${\boldsymbol{H}}_{\boldsymbol{corr}}^{0\to 298.15}$ **calc.**	6.20	6.20	4.34	4.34	4.34	4.34

where D_0_ is the dissociation energy which is equal to the atomization energy for a number of x atoms of the type X in molecule M. We compared the selected methods by comparing thermodynamic data of the BeH and the BeH_2_ molecules with experimental data from the NIST-JANAF database [49]. The results are shown in Table 2. All methods predict values of ∆_f_H^°^(BeH) rather close to the experimental value of 321 ± 30 kJ mol^-1^ (= 3.3 ±0.3 eV) [49], with CCSD(T) differing the most by ∼19 kJ mol^-1^ (0.2 eV). ∆_f_H^°^ (BeH_2_) is significantly overestimated compared to the experimental value of ∼125.52 kJ mol^-1^ (1.3 eV) which is based on an empirical method [49, 50]. For BeH_2_, the DFT functionals overall give values closer to the experimental value than the higher order methods, which differ by ∼33–43 kJ mol^-1^ (0.3–0.5 eV). The M06 functional predicts enthalpies of formation closest to the reference values. B3LYP-D and CCSD(T) harmonic frequencies are closest to the experimental data. The harmonic frequency for BeD obtained with B3LYP-D and CCSD(T) methods, 1530.4 cm^−1^, agrees well with the experimental value of 1529.5 cm^−1^ [32].

**Table 2:** Comparison of calculated thermodynamic properties as obtained by various methods for BeH, BeH_2_, and H_2_(D_2_) molecules to known experimental values. ∆_*f*_ *H* ^°^ is in kJ mol^-1^, R_e_ in Å, and ω_e_ in cm^−1^. The enthalpy of formation for BeH and its estimation for BeH_2_ are taken from ref. [50]. Experimental values for the hydrogen molecule are taken from the NIST-JANAF thermochemical Tables [49]. Spectroscopic values for BeH and BeH_2_ are retrieved from ref. [32, 33]. ${\omega}_e^a$ for BeH_2_ corresponds to the asymmetric stretching vibration and ${\ \omega}_e^b$ to the bending vibration

**Method**		**BeH**		**BeH**_**2**_				**H**_**2**_		**D**_**2**_
	∆_***f***_ ***H*** ^°^	***R***_***e***_	***ω***_***e***_	∆_***f***_ ***H*** ^°^	***R***_***e***_	${\boldsymbol{\omega}}_{\boldsymbol{e}}^{\boldsymbol{a}}$	${\boldsymbol{\omega}}_{\boldsymbol{e}}^{\boldsymbol{b}}$	***R***_***e***_	***ω***_***e***_	***ω***_***e***_
**B3LYP-D**	307.86	1.340	2062.4	133.7	1.324	2263.5	724.8	0.742	4415.2	3123.2
**B97D**	328.60	1.367	1936.8	140.98	1.337	2201.8	706.0	0.744	4373.5	3093.7
**B2PLYPD**	316.65	1.338	2084.9	144.90	1.323	2278.4	726.8	0.740	4461.2	3155.8
**M06**	313.34	1.341	2089.0	128.77	1.326	2264.6	705.2	0.744	4423.8	3129.3
**G4**	331.52	1.344	2064.5	158.93	1.327	2275.6	746.3	0.743	4465.7	3159.0
**CCSD(T)**	340.23	1.342	2062.4	168.31	1.327	2257.2	717.1	0.742	4401.1	3113.2
**Exp.**	321 ± 30	1.342	2061.4	125.52	1.326	2255.2	706.3	0.741	4401.2	3115.5

Eventually, to obtain the total change of the free energy of reaction, a term dependent on initial concentrations or more generally on instantaneous activities ${a}_j^{v_j}$ of products and ${a}_i^{v_i}$ of reactants, referred to as reaction quotient, *Q*_*r*_, has to be added [48]:

4 $$ {\varDelta}_r\mathrm{G}\left(\mathrm{T}\right)={\varDelta}_r{G}^{{}^{\circ}}(T)+ RT\mathit{\ln}{Q}_r= RTln\frac{Q_r}{K_{EQ}} $$

5 $$ {Q}_r=\frac{\prod_j{a}_j^{v_j}(t)}{\prod_i{a}_i^{v_i}(t)} $$

The ratio *Q*_*r*_ / *K*_*EQ*_ determines the direction of reaction: if *Q*_*r*_ > *K*_*EQ*_, the reaction favors the reactants; if *Q*_*r*_ < *K*_*EQ*_, the products are preferable. The reaction is in equilibrium for *Q*_*r*_ = *K*_*EQ*_. The reaction quotients were not calculated nor otherwise included for studied dissociation channels in this work.

### Localizing transition states and calculating rate constant

Approximate transition states geometries were at first guessed instead of using computational methods such as QST2 [51]. These structures were then optimized using the B3LYP-D functional. It was checked by vibrational frequency analysis that a transition state has only one imaginary frequency, with modes corresponding to the reaction path. In addition, IRC calculations were performed to ensure that the obtained transition states connect the local minima on the PES which refer to reactants and products for the considered reactions. Subsequently, obtained structures were optimized by the CCSD(T) method. The rate constants were determined only for dissociation channels with log(K_EQ_) > −5 for any point in the temperature range from 0 to 1000 K because those are the reactions most affecting plasma-wall interactions. In case of reactions with log(K_EQ_) < −5 nearly only reactants will appear in the equilibrium mixture [48]. We used transition state theory (TST) to estimate the reaction rate constants for temperature T by employing the Eyring–Polanyi Eq. [52]:

6.a $$ k(T)=\frac{k_B T}{h}\ \frac{1}{c^n\ }{e}^{\frac{\varDelta {G}^{\ddagger }(T)}{RT}} $$

6.b $$ \mathit{\ln}\frac{k(T)}{T}=\frac{-\varDelta {H}^{\ddagger }}{R}\ \frac{1}{T}+\mathit{\ln}\frac{k_B}{h}+\frac{{\varDelta S}^{\ddagger }}{R} $$

where k_B_ is the Boltzmann constant, R the gas constant, h the Planck constant, c the concentration, n the order of reaction, and ΔG^‡^ is the free energy of activation. We set c to 1 for results in the present work. The linear form of this equation (Eq. 6.b), where ∆*H* ^‡^ and ∆*S* ^‡^ are the enthalpy and entropy of activation, was used to present the calculated rate constants. From the rate constants, we are able to determine the reaction schemes for unmixed reactants and products in their standard states at the pressure of 1 atm and thus can predict the feasibility of the studied reactions based on the data from computational electronic structure methods and the rules of chemical kinetics.

### Molecular dynamics simulations

**Fig. 1:**
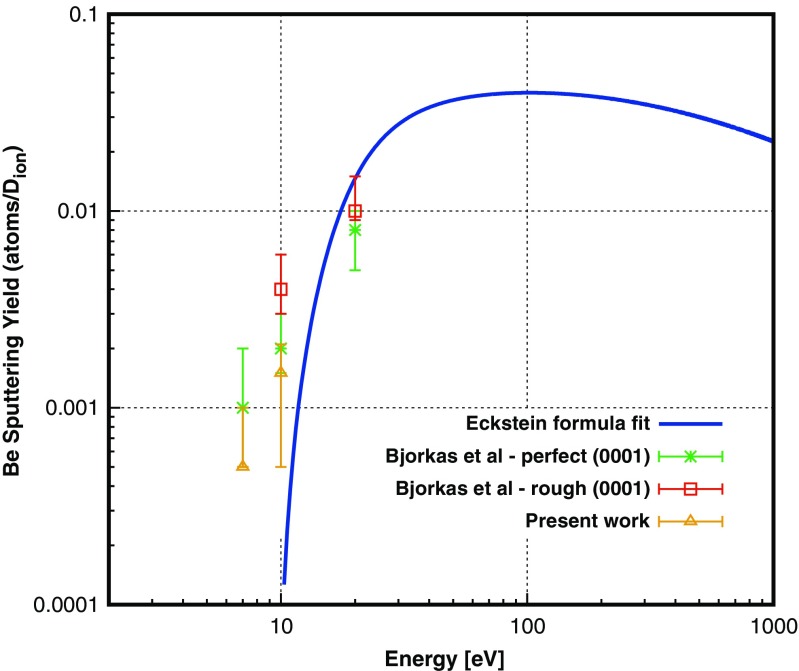
Sputtering yields obtained from MD simulations of D irradiated Be surfaces compared with literature data [15]

We studied the sputtering of BeD by low energy D irradiation from pure Be surfaces by means of molecular dynamics (MD) simulations using the same procedure as in ref. [15]. The D^+^ bombardment was simulated with the DL_POLY 3.9 software [53] which was extended to include ABOP potentials [54]. The details and parameters of the Be-H potentials are given in ref. [17]. The hexagonal closed packed Be surface (0001) with 3718 atoms (30×30×40 Å) was equilibrated by slowly heating the samples to 300 K at a rate of 50 K/ps. Subsequently, 1000 cumulative D impacts with 7, 10, and 20 eV were performed from a distance of 5 Å perpendicular to the center of the surface. A single impact lasted 7 ps and was divided into two parts: the first 3 ps consist of the impact itself followed by 4 ps of relaxation of the cell to remove extra energy from the system. Each step lasted 0.5 fs. The surface was randomly shifted in x- and y-directions after each impact. We compared the sputtering yields with other work (see Fig. 1) and extracted data about single sputtering events to look closer at the mechanisms.

## Results and discussion

### Stability analysis

We obtained optimized geometries of neutral and charged BeH, BeH_2_, and BeH_3_ molecules from the various functionals, the G4, and CCSD(T) methods. Concerning BeH_2_ the optimized structures all have negative electron affinities with an absolute value of 5–32 kJ mol^-1^ (0.1–0.3 eV), i.e., an energy is required to attach an electron. They are thus thermodynamically unstable and were removed from further analysis. Be, H, and H_2_ are also included in this analysis. The structural properties of H_2_, BeH, and BeH_2_ are given in Table 2, the ones for the remaining molecules are summarized in Table 3. Dissociation energies and enthalpies of formation for neutral and positive ions of beryllium deuterides are given in Tables 4 and 5, respectively.

**Table 3:** Bond lengths and angles of the beryllium hydrides. The lengths are given in Å, angles in degrees. For the meaning of R_1_ and R_2_ see Fig. 3. The data for BeH and BeH_2_ are given in Table 2

	**BeH** ^**+**^	**BeH** ^**−**^	**BeH**_**2**_^**+**^ **I**		**BeH**_**2**_^**+**^ **II**		**BeH**_**3**_		**BeH**_**3**_^**+**^		**BeH**_**3**_^**−**^
	*R*_*e*_	*R*_*e*_	*Be-H*	*H-Be-H*	*Be-H*	*H-Be-H*	*R*_*1*_	*R*_*2*_	*R*_*1*_	*R*_*2*_	*R*_*1*_
**B3LYP-D**	1.313	1.408	1.411	93.3	1.877	23.6	1.322	1.417	1.299	1.663	1.415
**B97D**	1.324	1.424	1.427	99.2	1.921	23.1	1.333	1.432	1.311	1.706	1.430
**B2PLYPD**	1.310	1.407	1.403	86.5	1.403	86.5	1.321	1.415	1.297	1.655	1.413
**M06**	1.307	1.397	1.404	89.5	1.883	23.5	1.322	1.421	1.298	1.693	1.415
**G4**	1.320	1.477	1.416	94.6	1.855	23.8	1.324	1.418	1.305	1.657	1.408
**CCSD(T)**	1.311	1.412	1.399	80.1	1.817	24.3	1.324	1.416	1.299	1.651	1.418

**Table 4:** Dissociation energy D_0_ and enthalpy of formation for the neutral beryllium deuterides. The electron affinity EA of BeH and BeH_3_ are also listed. All values are in kJ mol^-1^

**Method**	**BeD**		**BeD**_**2**_		**BeD**_**3**_		**BeH= > BeH** ^**−**^	**BeH**_**3**_ **= > BeH**_**3**_^**−**^
	**D**_**0**_	∆_***f***_ ***H*** ^°^	**D**_**0**_	∆_***f***_ ***H*** ^°^	**D**_**0**_	∆_***f***_ ***H*** ^°^	**EA**	**EA**
**B3LYP-D**	233.6	308.4	625.6	132.8	680.5	295.8	50.9	275.1
**B97D**	212.7	329.3	618.2	140.3	668.4	307.9	70.9	273.1
**B2PLYPD**	207.4	334.6	597.0	161.4	633.6	342.6	39.4	267.8
**M06**	224.9	317.1	614.5	143.9	658.4	317.7	44.6	273.1
**G4**	228.2	313.8	630.6	127.9	661.4	314.9	63.0	286.2
**CCSD(T)**	198.1	340.7	591.0	167.4	628.1	348.1	51.8	278.0

**Table 5:** Dissociation energy D_0_ and enthalpy of formation for the cationic beryllium deuterides. All values are in kJ mol^-1^

**Method**	**BeD** ^**+**^		**BeD**_**2**_^**+**^ **I**		**BeD**_**2**_^**+**^ **II**		**BeD**_**3**_^**+**^	
	**D**_**0**_	∆_***f***_ ***H*** ^°^	**D**_**0**_	∆_***f***_ ***H*** ^°^	**D**_**0**_	∆_***f***_ ***H*** ^°^	**D**_**0**_	∆_***f***_ ***H*** ^°^
**B3LYP-D**	292.7	395.7	395.7	1148.8	482.6	1177.2	831.8	1045.1
**B97D**	316.8	414.3	414.3	1124.7	483.5	1776.4	853.6	1023.5
**B2PLYPD**	301.3	361.5	361.5	1140.2	472.8	1189.7	835.8	1041.0
**M06**	289.6	363.3	363.3	1151.9	465.8	1195.0	822.2	1054.8
**G4**	306.6	379.5	379.5	1134.9	477.1	1182.4	832.8	1044.3
**CCSD(T)**	296.8	370.5	370.5	1144.7	471.1	1188.1	828.7	1048.2

**Fig. 2:**
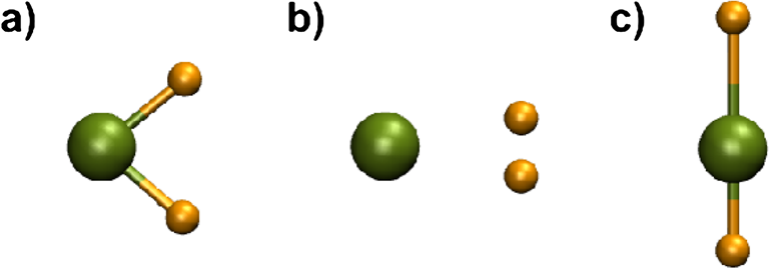
Local minima of cationic BeD_2_ obtained with CCSD(T). a-c refer to the structures I, II, and III in the text

**Fig. 3:**
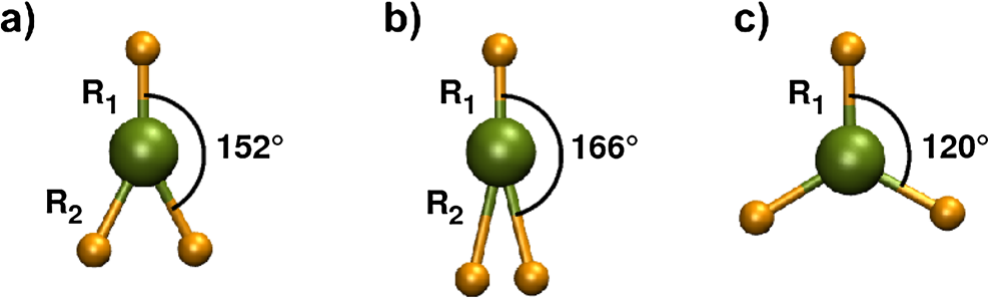
Structures of a) neutral, b) cationic, and c) anionic BeD_3_ obtained with B3LYP-D

The bond lengths for BeH range from 1.338 to 1.367 Å, with CCSD(T) and M06 being closest to the experimental value of 1.342 Å [32]. The bond length of the respective cation is shorter by 0.03 ± 0.01 Å on average, whereas the bond length of the respective anion is longer by 0.08 ± 0.03 Å. The Be-H bond lengths of neutral BeH_2_ range from 1.323 to 1.337 Å. Again, the CCSD(T) and M06 values agree excellently with the experimental bond length of 1.326 Å [33]. All methods predict two bent structures for ${\mathrm{BeH}}_2^{+}$, with H-Be-H angles of ∼90° (I) and ∼24° (II), and a multiple saddle point for the symmetric linear structure (Be-H: ∼1.46 Å). The bent structure (II) corresponds to the global minimum. Furthermore, only CCSDT(T) predicts another local minimum (III) for the asymmetric linear structure (Be-H: 1.323 Å, 1.716 Å). Their structures are depicted in Fig. 2. This is in contrast with the potential energy surface of ${\mathrm{BeH}}_2^{+}$ produced at CMRCI/cm^3^-pVTZ level of theory in ref. [29], where only the linear asymmetric structure is reported beside the van der Waals minimum. However, only ${\mathrm{BeH}}_2^{+}\ \mathrm{II}$ is below the dissociation limit for Be^+^ + H_2_. Furthermore, we optimized the equilibrium geometries of the other local minima for BeH_2_ and ${\mathrm{BeH}}_2^{+}$ of the same publication [29] with B3LYP-D and CCSD(T) to compare and validate our approach for transition state search. The the results are in good agreement (see Table 6), except for the fact mentioned above concerning the non-existence of an asymmetric linear structure of ${\mathrm{BeH}}_2^{+}$ for B3LYP-D. All methods yield similar structures for neutral and ionic BeH_3_ molecules (see Fig. 3). The angle formed between H-Be-H is notably different with ∼152° for neutral molecules, ∼166° for cations and 120° for anions. The experimental BeD bond length and bond length in BeD_2_ are 1.342 and 1.326 Å, respectively [32, 33].

**Table 6:** Bond lengths and angles of corresponding optimized structures obtained by B3LYP-D and CCSD(T) methods compared with data from ref. [29]

	**BeH**_**2**_ **linear**	**BeH**_**2**_ **(** ^**3**^ **B**_**2**_ **)**		**BeH**_**2**_^**+**^ **linear**		**BeH**_**2**_^**+**^ **(** ^**2**^ **A**_**1**_ **)**	
	*Be-H*	*Be-H*	*H-Be-H*	*R*_*1*_	*R*_*2*_	*R*_*1*_	*H-Be-H*
**B3LYP-D**	1.324	1.433	42.3	--	--	1.877	23.6
**CCSD(T)**	1.327	1.438	39.7	1.323	1.716	1.817	24.3
**CASSCF**	1.330	1.445	40.0	1.320	1.731	1.798	24.6
**CMRCI**	1.330	1.442	39.8	1.327	1.723	1.794	24.7

The dissociation energy D_0_ for BeH is in the range from ∼198 to 231 kJ mol^-1^ (2.0–2.4 eV) which agrees with experimental values of 221 ± 30 kJ mol^-1^ (2.3 ± 0.3 eV) reported by Gaydon [48], 208.4 ± 1.0 kJ mol^-1^ (2.16 ± 0.01 eV) by Colin [50], or the estimation of 230 kJ mol^-1^ (2.4 eV) in ref. [55]. BeD_2_ and BeD_3_ yield considerably higher atomization energies of 591–631 kJ mol^-1^ (6.1–6.5 eV) and 628–680 kJ mol^-1^ (6.4–7.1 eV). A similar trend is seen for positive ions, with 290–317 kJ mol^-1^ (3.0–3.3 eV), 362–414 kJ mol^-1^ (3.6–4.3 eV), 822–855 kJ mol^-1^ (8.5–8.9 eV) for BeD^+^, ${\mathrm{BeD}}_2^{+}$ I, and ${\mathrm{BeD}}_3^{+}$, respectively. We did not calculate D_0_ and enthalpies of formation of negative ions. The electron affinities (EA) of BeH and BeH_3_ were calculated (see Table 4). BeH (BeH_3_) gains about 50 (275) kJ mol^-1^ by electron attachment to form BeH^−^ (BeH_3_^−^). The latter value agrees excellently with the calculations in ref. [35]. There is data for photodetachment of BeH^−^ forming BeH_2_ via the reaction BeH^−^ + H^+^ → BeH_2_ [56]. The authors measured the enthalpy of reaction to be 1630 ± 13 kJ mol^-1^ which agrees with our calculated value of 1640–1665 kJ mol^-1^. The standard enthalpies of formation are similar for BeD and BeD_3_ yielding about 300–350 kJ mol^-1^ (3.1–3.6 eV). They are higher than the one for BeD_2_, for which ∆_f_H^°^ is about 130–170 kJ mol^-1^ (1.4–1.8 eV). The enthalpies of formation of beryllium deuterides are higher than for beryllium hydrides by ∼3 to 10 kJ mol^-1^ (and almost the same numbers apply for cations). Overall, B3LYP-D results in higher dissociation energies than CCSD(T). This is not the case for the enthalpies of formation. The B2PLYPD functional yields values that are very close to those obtained with CCSD(T).

### Thermodynamics

**Fig. 4:**
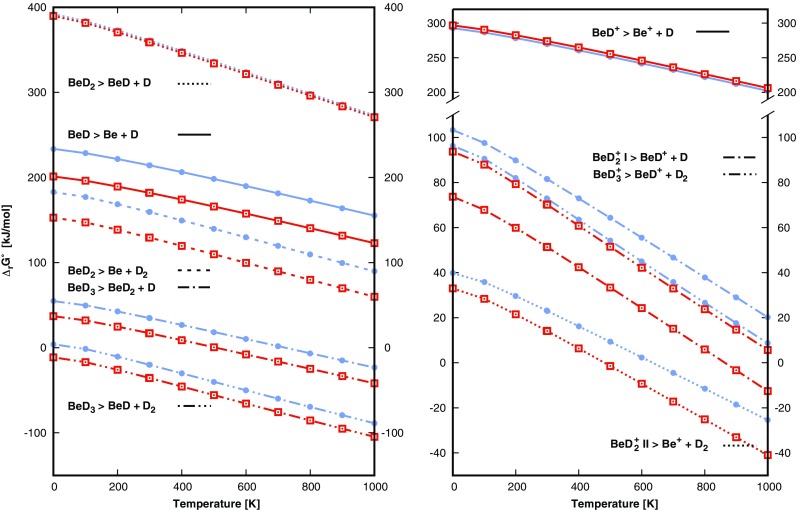
Changes of the standard free energies of reaction for a) neutral and b) positive beryllium deuterides given by B3LYP-D (full circle) and CCSD(T) (empty square) methods

Standard enthalpies ∆_r_H^°^ and the free energies ∆_r_G^°^ of reaction were calculated for all possible dissociation channels of the stable neutral and ionic beryllium deuterides in the temperature range from 0 to 1000 K. We selected reaction channels (Eqs. 7 and 8) with log(K_EQ_) > −5 for a further analysis of reaction pathways. The temperature dependences of the free energies of reaction are shown in Fig. 4 for the dissociation of neutral and cationic beryllium deuterides as calculated with B3LYP-D and CCSD(T). We calculated the enthalpies and the free energies of reactions for BeD and BeD^+^ based on very accurate data extracted from MR ACPF calculations by Koput [22] which serve as benchmark values for our results. ∆_r_H^°^ at 298.15 K and ∆_r_G^°^ at 298.15 K and 1000 K obtained by different methods are provided for neutral molecules and cations in Tables 7 and 8, respectively. The MR ACPF enthalpies and free energies of the reactions 7.a and 8.a are very close to G4 and CCSD(T) values yielding differences up to 5 kJ mol^-1^ for G4 and CCSD(T) indicating that BeD and BeD^+^ do not yield strong multi-referential character of the wave functions in equilibrium. However, this will be discussed in more detail in the next section which is dedicated to transition states.

**Table 7:** Changes of the enthalpy of reaction at 298.15 K and changes of the free energy of reaction at 298.15 and 1000 K for the dissociation of neutral beryllium deuterides obtained from various methods. MR ACPF values are calculated from data in ref. [22]. All values are in kJ mol^-1^. The threshold temperature T_k_ for crossing the log(K_EQ_) > −5 limit is also given

**Method**	***BeD***(***g***)→ ***Be***(***g***) + ***D***(***g***)			***BeD***_2_(***g***)→ ***Be***(***g***) + ***D***_2_(***g***)			***BeD***_2_(***g***)→ ***BeD***(***g***) + ***D***(***g***)			***BeD***_3_(***g***)→ ***BeD***(***g***) + ***D***_2_(***g***)			***BeD***_3_(***g***)→ ***BeD***_2_(***g***) + ***D***(***g***)		
	∆_***r***_ ***H*** ^°^	∆_***r***_ ***G*** ^°^		∆_***r***_ ***H*** ^°^	∆_***r***_ ***G*** ^°^		∆_***r***_ ***H*** ^°^	∆_***r***_ ***G*** ^°^		∆_***r***_ ***H*** ^°^	∆_***r***_ ***G*** ^°^		∆_***r***_ ***H*** ^°^	∆_***r***_ ***G*** ^°^	
**T[K]**		298.15	1000		298.15	1000		298.15	1000		298.15	1000		298.15	1000
**B3LYP-D**	237.3	214.4	155.2	188.0	159.5	89.8	397.3	361.2	273.0	9.5	−20.0	−88.8	58.7	35.0	-23.4
**B97D**	216.4	193.6	134.7	174.0	145.6	76.3	410.7	374.6	286.5	11.7	−17.6	−85.9	54.1	30.5	-27.4
**B2PLYPD**	228.6	205.67	146.4	183.5	154.9	85.2	394.9	358.8	270.5	2.8	−26.8	−96.1	47.9	24.0	-34.8
**M06**	231,9	209.0	149.7	202.6	174.1	104.6	407.6	371.6	283.7	5.4	−23.9	−92.1	34.7	11.0	-47.0
**G4**	211,0	188.1	128.9	161.1	132.5	62.5	395.0	358.8	270.3	−9.8	−39.4	−108.8	40.1	15.4	-43.7
**CCSD(T)**	204.5	182.1	122.8	158.0	129.5	59.9	395.0	358.9	270.8	−5.9	−35.5	−104.8	31.0	17.1	-41.8
**MR ACPF**	206.6	186.4	131.9	---	---	---	---	---	---	---	---	---	---	---	---
**T**_**k**_ **[K]**	**---**			∼800			**---**			already at 0			∼200

**Table 8:** Changes of the standard enthalpy of reaction at 298.15 K and changes of the standard free energy of reaction at 298.15 and 1000 K for dissociation of cationic beryllium deuterides obtained from various methods. MR ACPF values are calculated from data in ref. [23]. All values are in kJ mol^-1^. The values for the bent structure of ${\mathrm{BeD}}_2^{+}$ are used here. The threshold temperature T_k_ for crossing log(K_EQ_) > −5 limit is also presented

**Method**	***BeD*** ^+^(***g***) → ***Be*** ^+^(***g***) + ***D***(***g***)			${\boldsymbol{Be}\boldsymbol{D}}_2^{+}\left(\boldsymbol{g}\right)\ \boldsymbol{II}\to {\boldsymbol{Be}}^{+}\left(\boldsymbol{g}\right)+{\boldsymbol{D}}_2\left(\boldsymbol{g}\right)$			${\boldsymbol{BeD}}_2^{+}\left(\boldsymbol{g}\right)\ \boldsymbol{I}\to \boldsymbol{Be}{\boldsymbol{D}}^{+}\left(\boldsymbol{g}\right)+\boldsymbol{D}\left(\boldsymbol{g}\right)$			${\boldsymbol{BeD}}_3^{+}\left(\boldsymbol{g}\right)\to \boldsymbol{Be}{\boldsymbol{D}}^{+}\left(\boldsymbol{g}\right)+{\boldsymbol{D}}_2\left(\boldsymbol{g}\right)$		
	∆_***r***_ ***H*** ^°^	∆_***r***_ ***G*** ^°^		∆_***r***_ ***H*** ^°^	∆_***r***_ ***G*** ^°^		∆_***r***_ ***H*** ^°^	∆_***r***_ ***G*** ^°^		∆_***r***_ ***H*** ^°^	∆_***r***_ ***G*** ^°^	
**T[K]**		298.15	1000		298.15	1000		298.15	1000		298.15	1000
**B3LYP-D**	296.4	269.9	202.2	43.3	23.1	−25.5	106.5	81.2	19.4	99.8	70.5	2.0
**B97D**	320.5	294.1	226.5	37.4	16.2	−35.0	101.2	75.9	13.9	91.5	64.4	1.5
**B2PLYPD**	293.0	266.5	198.7	38.7	16.4	−37.8	92.2	66.4	3.2	99.7	72.0	7.4
**M06**	310.3	283.9	216.1	36.4	14.2	−39.8	84.5	58.5	−5.6	97.1	70.0	7.0
**G4**	304.8	278.4	210.8	39.6	−17.5	−35.9	76.0	50.9	−10.6	97.3	69.4	4.4
**CCSD(T)**	300.5	274.1	206.3	36.9	−14.2	−41.0	77.6	51.5	−12.6	98.0	70.3	5.7
**MR ACPF**	301.9	278.9	215.5	---	---	---	---	---	---	---	---	---
**T**_**k**_ **[K]**	**---**			∼300			∼400			∼500

7.a $$ Be D(g)\to Be(g)+ D(g) $$

7.b $$ {BeD}_2(g)\to Be(g)+{D}_2(g) $$

7.c $$ {BeD}_2(g)\to BeD(g)+ D(g) $$

7.d $$ {BeD}_3(g)\to BeD(g)+{D}_2(g) $$

7.e $$ {BeD}_3(g)\to {BeD}_2(g)+ D(g) $$

8.a $$ {Be D}^{+}(g)\to {Be}^{+}(g)+ D(g) $$

8.b $$ {Be D}_2^{+}(g)\  II\to {Be}^{+}(g)+{D}_2(g) $$

8.c $$ {BeD}_2^{+}(g)\  I\to {BeD}^{+}(g)+ D(g) $$

8.d $$ {BeD}_3^{+}(g)\to {BeD}^{+}(g)+{D}_2(g) $$

Almost all studied dissociation channels have equilibrium constants very close to 0 (K_EQ_ < < 1); the reactants dominate in the mixtures and an increase of the concentration of products leads to the production of more reactants. Reaction 7.c has a rather high ∆_r_H^°^ value of 395 kJ mol^-1^ for G4 and 411 kJ mol^-1^ for B97D (4.1–4.3 eV) at 298.15 K. BeD_2_ more likely dissociates into Be and D_2_ with ∆_r_H^°^ (298.15 K) = 188.0 kJ mol^-1^ for B3LYP-D. The change of standard free energy predicts log(K_EQ_) > −5 for more than ∼800 K. For BeD_3_, both channels (7.d and 7.e) have similar characteristics with ∆_r_G^°^ (T) close to 0 kJ mol^-1^ already at 0 K. ∆_r_G^°^ (T) for dissociation of BeD lies in the range of 203–237 kJ mol^-1^ (2.1–2.5 eV) at 298.15 K. BeD^+^ has the highest ∆_r_G^°^ (T) of the reported cations. 8.b is the preferable channel of the two most likely ways of the dissociation of ${\mathrm{BeD}}_2^{+}$ (∆_r_G^°^ < 0 already at 300 K). The second channel, reaction 8.c, has a lower ∆_r_H^°^ than its neutral alternative: 76–106 kJ mol^-1^ at 298.15 K (0.7–1.1 eV) with log(K_EQ_) **< −**5 at ∼500 K. There is only one channel (8.d) with ∆_r_H^°^ = 91–100 kJ mol^-1^ (0.9–1.0 eV) for ${\mathrm{BeD}}_3^{+}$ as the others all have a log(K_EQ_) **< −**5. This reaction has log(K_EQ_) > −5 from ∼500 K on.

### Reactivity of beryllium deuterides

**Fig. 5:**
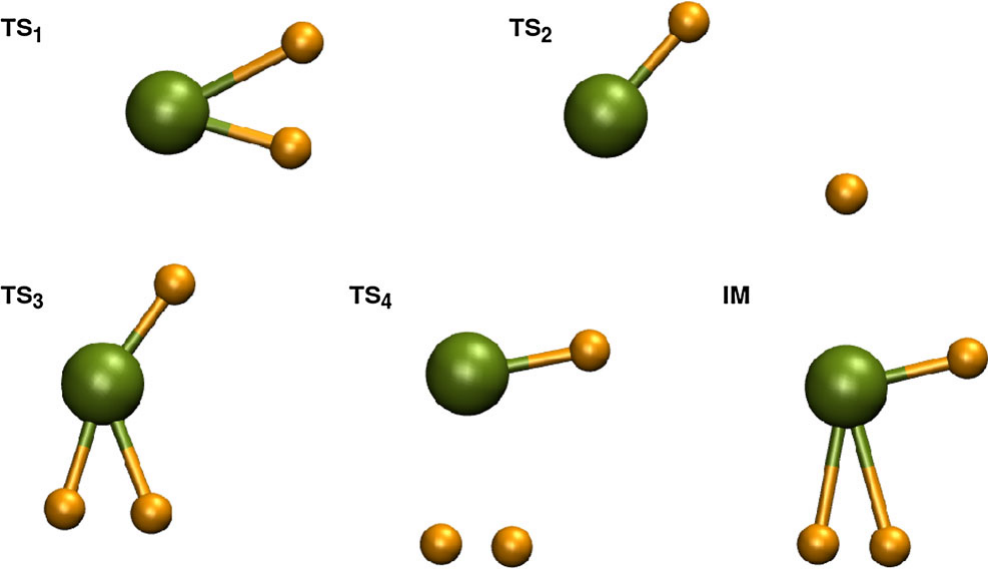
Structures of the transition states for dissociation channels 7.b-d

**Fig. 6:**
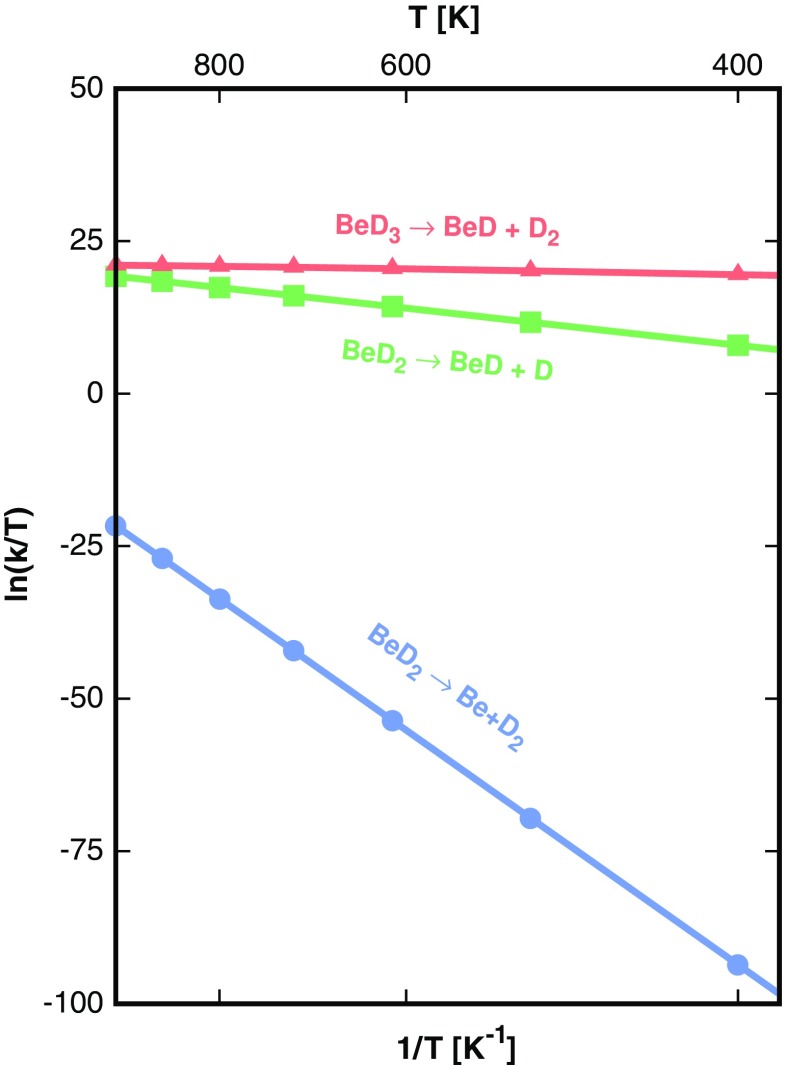
Dependence of reaction rates on temperature in the linear form of Eyring equation (Eq. 6.b) for channels with identified transition states

The structural properties and the free energy of activation ΔG^‡^ of transition states obtained using the CCSD(T) method for the forward and reverse reactions along each channel are presented in Tables 9 and 10, respectively. Their structures are depicted in Fig. 5. Reaction rate constants can be calculated using Eq. 6. The rate constants in the linear form of the Eyring-Polanyi equation for channels with identified transition states of Eqs. 7 are plotted in Fig. 6.

**Table 9:** Bond lengths and angles of the transition states obtained by CCSD(T) for studied dissociation channels in Eqs. 7 and 8. The lengths are given in Å, angles in degrees. These structures are presented in Fig. 5

	**BeH**_**2**_		**BeH**_**3**_		
	*TS*_*1*_	*TS*_*2*_	*TS*_*3*_	*TS*_*4*_	*IM*
Be-H1	1.379	1.344	1.323	1.340	1.339
Be-H2	1.646	2.827	1.403	1.852	1.714
Be-H3	---	---	1.498	1.917	1.776
H1-Be-H2	43.5	67.5	161.2	109.5	113.5
H1-Be-H3	---	---	123.4	86.3	87.9

**Table 10:** Activation energies ΔG^‡^ corresponding to the transition states TS for the dissociation channels of the beryllium deuterides at 298.15 K, 600 K, and 1000 K for the forward and reverse reactions obtained by the CCSD(T) method

	Transition state	Forward			Reverse		
		ΔG^‡^ [kJ mol^-1^]			ΔG^‡^ [kJ mol^-1^]		
T [K]		298.15	600	1000	298.15	600	1000
*BeD*_2_(*g*) → *Be*(*g*) + *D*_2_(*g*)	TS_1_	392.6	385.8	377.8	263.1	286.0	317.9
*BeD*_2_(*g*) → *BeD*(*g*) + *D*(*g*)	TS_2_	377.4	362.9	344.6	18.5	41.4	73.8
*BeD*_3_(*g*) → *BeD*(*g*) + *D*_2_(*g*)	TS_3_	13.4	16.3	22.4	48.9	82.0	127.2
	TS_4_	-0.5	−1.3	−0.6	34.9	64.4	104.2

#### BeD and BeD^+^

We did not find any transition state concerning the dissociation of BeH or BeH^+^ in line with earlier studies [22–24].

#### BeD_2_ and ${BeD}_2^{+}$

A more complex behavior is found for the reactivity of neutral and cationic BeD_2_. Beryllium dihydride can dissociate into Be + D_2_ through the transition state TS_1,_ which corresponds to the one found in ref. [29]. No transition state was found for the other channel (7.c) in the ground state, but we localized one (TS_2_) in the triplet state. CCSD(T) yields the same transition state structures as B3LYP-D. Both transition states are very close in energy and in the region where the ground state potential energy surface intersects the one of the triplet state. Therefore, their activation free energies are similar and rather high, ∼380 kJ mol^-1^ (3.9 eV), with regard to the BeD_2_ ground state. TS_2_ is about 55 kJ mol^-1^ (0.6 eV) higher than the triplet state local minima and about 30 kJ mol^-1^ (0.3 eV) below the plateau of the excited Be + D_2_ complex. We assume that the channel resulting in Be + D_2_ is preferable in general, however, in a fusion plasma environment the required excitation energy is easily reachable and dissociation into BeD + D is therefore also possible. The difference of ΔG^‡^ for the two transition states, ∼20 kJ mol^-1^ (0.2 eV) in the range 0–1000 K, favors decomposition into BeD + D. The reverse reaction 7.b has a high barrier as well, ∼260 kJ mol^-1^ (2.7 eV) at 298.15 K and ∼320 kJ mol^-1^ (3.3 eV) at 1000 K. We could not identify any transition state for channels 8.b and 8.c with CCSD(T). Still, ${\mathrm{BeD}}_2^{+}$ is predicted to dissociate into Be^+^ + D_2_ due to the negative free reaction energy already at 300 K.

#### BeD_3_ and ${\mathrm{BeD}}_3^{+}$

The BeD_3_ molecule is metastable. We found a two-step reaction mechanism for reaction 7.d. However, the intermediate (IM) and the transition state TS_4_ (see Fig. 4) are very close in energy and similar in structure and are also lower in energy than the local minima at higher temperatures. Thus, they make the decomposition into BeD + D_2_ more likely than the competing reaction 7.e. The free activation energies, ΔG^‡^, for these barriers are ∼14 kJ mol^-1^ (0.1 eV) at 298.15 K and ∼ −0.5 kJ mol^-1^ (0.01 eV) at 298.15 for TS_3_ and TS_4_, respectively. B3LYP-D predicts the same characteristics of the transition states as CCSD(T). We did not identify any transition state for the reaction in Eq. 8.d. In fact, BeD_3_^+^ seems to dissociate most likely into BeD^+^ + D_2_ as this channel is energetically preferable (∆_r_G^°^ ∼0 at 1000 K).

The transition states found using B3LYP-D and CCSD(T) were also investigated for their multi-referential character using the D_1_ and T1 diagnostics [57, 58]. The results are in excellent agreement with those of higher-order correlation methods if D_1_ < 0.03. If D_1_ < 0.05, the method still performs well. However, a multi-reference character of the ground-state introduced by strong orbital relaxation effects is indicated by larger values of D_1_. Similarly, if T_1_ > 0.02, the system should be investigated by a multi-reference electron correlation method. The conclusion of the D_1_ and T_1_ diagnostics for BeH molecules is as follows: all equilibrium structures have D_1_ less than 0.03 and T_1_ < 0.02, except asymmetric linear ${\mathrm{BeD}}_2^{+}$ (T_1_ = 0.02,D_1_ = 0.08), thus CCSD(T) should describe their ground states reliably. Far from equilibrium and for some transition states these diagnostics yield higher values, indicating that single-reference methods could become inadequate for describing these states. This concerns only transition states for neutral and positive BeH_2_ (T_1_ ∼0.35, D_1_ ∼0.08). Transition states related to BeH_3_ molecules yield D_1_ < 0.03, with minimum and maximum values of 0.015 and 0.029, respectively, and T_1_ ∼0.01. Further investigations are required to scrutinize how the various PESs and energetics are affected by more accurate correlation and multi-referential character, which we plan to do in a following work.

#### Production of BeD

Analysis of our MD simulations yields that sputtering of BeD can often be described by the following reactions (9.a and 9.b) ignoring the surrounding surface atoms. BeD_2_ and BeD_3_ turn out to be intermediate products that last only for a few steps.

9.a $$ Be{D}_2(g)+ D(g)\to {\  BeD}_3(g)\to Be D(g)+{D}_2(g) $$

9.b $$ Be(g)+{D}_2(g)\to {BeD}_2(g)\to Be D(g)+ D(g) $$

The reaction 9.b without the intermediate BeD_2_ was also suggested and analyzed by Nishijima et al. [55]. However, the ionic reaction Be^+^ + D_2_ was shown to dominate the production of BeD^+^ inside a plasma column. Based on our calculated standard free energies of reaction and reaction schemes for BeD_2_ and BeD_3_ we can determine the reactivity of the proposed reactions in Eq. 9. BeD_2_ and D can easily form the BeD_3_ intermediate dissociating subsequently into BeD + D_2_. Impinging particles with small kinetic energy could possibly lead to BeD and D_2_ via this reaction pathway. The other reaction, 9.b, has a high barrier for all studied temperatures, but it is a possible reaction nonetheless. Concerning the production of BeD from the surface, reaction 9.b is slower than 9.a and higher impact energies are needed.

## Conclusions

We report reaction schemes of the dissociation of small beryllium deuterides based on calculated thermodynamic properties, standard enthalpies of formation, and standard enthalpies and free energies of reaction as obtained by quantum-chemical methods. Transition state theory was used to determine the rate constants for the considered reactions. BeD and BeD^+^ are the most stable species, unlikely to further dissociate into their components. BeD_2_ and ${\mathrm{BeD}}_2^{+}$ are more likely to decompose into Be + D_2_ than into BeD + D. BeD_3_ and ${\mathrm{BeD}}_3^{+}$ are metastable against their dissociation into BeD + D_2_. Concerning the source of beryllium hydride production, we performed MD simulations of low energy D irradiation on Be surfaces to obtain the details of the sputtering events and analyzed these events from thermodynamic and kinetic points of view. The analysis of the MD trajectories confirms that the formation of BeD occurs along the reaction pathways that have been suggested before.
